# Inferior vena cava collapsibility index as a predictor of hypotension after induction of general anesthesia in hypertensive patients

**DOI:** 10.1186/s12871-023-02355-y

**Published:** 2023-12-19

**Authors:** Mohamed Metwaly Fathy, Rehab A Wahdan, Amal Abdul Azeem Salah, Abeer M Elnakera

**Affiliations:** Department of Anesthesia, Intensive Care, and Pain Management, Faculty of Medicine, Zagazig University, Zagazig, Egypt

**Keywords:** Inferior vena cava, Collapsibility index, Hypotension, General anesthesia, Hypertension

## Abstract

**Background:**

Hypertensive patients are more susceptible to develop hypotension after the induction of general anesthesia (GA), most likely due to hypovolemia. An inferior vena cava collapsibility index (IVCCI) > 40–50% can predict hypotension after the induction of GA in the general population by variable accuracies. The current study aimed to investigate IVCCI% as a predictor of postinduction hypotension in hypertensive patients undergoing noncardiac surgery.

**Methods:**

Ultrasound IVCCI % was assessed for all controlled hypertensive patients immediately before induction of GA. After induction of GA, patients were diagnosed with postinduction hypotension if their systolic arterial pressure (SAP) dropped by ≥ 30% of the baseline value and/or mean arterial pressure (MAP) dropped to < 65 mmHg up to 15 min after intubation. The receiver operating characteristic (ROC) curve of IVCCI% was compared to patients’ classification either developing hypotension after induction of GA or not as a gold standard.

**Results:**

Of the 153 patients who completed the study, 62 (40.5%) developed hypotension after the induction of GA, and 91 (59.5%) did not. An IVCCI > 39% predicted the occurrence of postinduction hypotension with high accuracy (84%) (AUC 0.908, *P* < 0.001). The area of uncertainty (by gray zone analysis) of IVCCI lies at values from 39 to 45%. This gray zone included 21 patients (13.7% of all patients).

**Conclusion:**

An inferior vena cava collapsibility index > 39% before anesthetic induction can be a simple noninvasive reliable predictor of hypotension after the induction of GA for hypertensive patients not treated with angiotensin-converting enzyme inhibitors (ACEIs) or angiotensin receptor blockers (ARBs) and undergoing noncardiac surgery.

**Trial registration:**

This clinical trial was approved by the Institutional Review Board (IRB) at Zagazig University (ZUIRB #9424 dated 03/04/2022), and patients’ informed consent for participation in the study was obtained during the period from May 2022 to May 2023. All study procedures were carried out in accordance with the ethical standards of the Helsinki Declaration of 2013.

## Background

Hypotension is a common complication in patients under general anesthesia since its incidence after general anesthesia induction ranges from 8 to 9% [1]. After induction of general anesthesia, patients are at particular risk of developing hypotension because of the cardiovascular depressant and vasodilatory effects of anesthetic agents, as well as lack of surgical stimulation. Furthermore, patients may have preexisting hypovolemia resulting from dehydration and impaired compensatory responses [2, 3].

Hypertensive patients are more likely to experience hemodynamic instability during general anesthesia (GA). The incidence of postinduction hypotension reaches 65% in hypertensive patients [4], which in turn maximizes the risk of postoperative adverse outcomes [5]. Hypovolemia is the most likely risk factor for postinduction hypotension, so the identification and management of latent hypovolemia can reduce the incidence of such complications [6]. The role of assessing intravascular volume status in the prediction of postinduction hypotension has not been fully investigated in hypertensive patients [7].

Several invasive tools (e.g., pulmonary arterial catheter) are available for evaluating preload among other elements of hemodynamic status, but their universal use is not a reasonable option due to financial constraints, relatively high complication rates, known limitations and unnecessary invasiveness compared to most surgical procedures [8].

Noninvasive ultrasound examination by anesthesiologists is a widespread and useful aid in the safe application of anesthesia and the evaluation of important aspects, such as global left ventricular function, ventricular diameters, pericardial effusion, or the diameter of the inferior vena cava [9]. Ultrasound assessment of the inferior vena cava collapsibility index (IVCCI) is an easy tool to assess fluid responsiveness in critically ill patients [10]. An IVCCI of 40–50% was shown to predict the onset of hypotension after the induction of GA with variable sensitivities and specificities in the general adult population [6, 11]. IVCCI could not reliably predict the onset of hypotension after induction of GA in patients scheduled for elective abdominal surgery [12]. This finding was similar to Vignon’s experience from critical care settings (IVC distensibility of ventilated patients) [13]. Nevertheless, IVCCI is the most validated tool for spontaneous breathing and the simplest to measure.

The aim of the current study was to investigate preoperative IVCCI% as a predictor of hypotension after induction of GA in hypertensive patients undergoing noncardiac surgery.

## Methods

This prospective, observational study was performed in the Department of Anesthesiaو Intensive Care and Pain Management, Faculty of Medicine, Zagazig University, after Institutional Review Board (IRB) approval (ZUIRB #9424 dated 03-04-2022) and obtaining patients’ informed consent for participation in the study during the period from May 2022 to May 2023. All study procedures were carried out in accordance with the ethical standards of the Helsinki Declaration of 2013.

Patients of both sexes who were aged 21–70 y, ASA II or III, had a medical history of controlled hypertension, had a body mass index (BMI) < 35 kg/m2 and underwent elective noncardiac surgery under GA were included in the study.

Exclusion criteria included patient refusal, suspected difficult airway, chronic obstructive pulmonary disease (COPD), elevated systolic pulmonary arterial pressure (> 40 mmHg), depressed left ventricular function (EF < 40%), severe congenital or valvular heart diseases, implanted pacemaker/cardioverter defibrillator, current angiotensin-converting enzyme inhibitors (ACEIs) or angiotensin receptor blockers (ARBs) medications, baseline systolic arterial pressure (SAP) ≥ 180 mmHg or < 90 mmHg, secondary hypertension, cerebrovascular or peripheral vascular disease, agitation (Richmond Agitation Sedation Scale (RASS) > + 1) [14], chronic renal failure on regular dialysis, pregnancy, ascites or increased intraabdominal pressure. Withdrawal criteria were poor ultrasound IVC visualization, prolonged intubation attempt (> 30 s) or cancellation of surgery.

All patients were thoroughly evaluated and medically optimized through the preoperative preparation outpatient clinic. The IVCCI measurement procedure was explained to the patient on the night of surgery. All patients were premedicated with 10 mg oral diazepam on the night of the surgery. Oral feeding was withheld for 8 h overnight. Clear fluids were allowed until 4 h before induction of GA. Regular antihypertensive medications were continued on their established routine, except for diuretics, which were withheld on the morning of surgery.

On patient arrival to the operating room, standard monitoring was applied, including 5-lead ECG, pulse oximetry and a noninvasive oscillometer for blood pressure monitoring (Datex-Ohmeda model (S/S) AN.S. NO:3,422,715, Finland,1998). Invasive arterial blood pressure monitoring was used by the attending anesthetist according to the invasiveness of the planned surgery and the risk level of the patient.

Before induction of GA, the IVC was examined while the patient had been spontaneously, quietly breathing and lying in the supine position for at least 5 min before assessment. A two-dimensional image of the IVC as it entered the right atrium was obtained through the paramedian long-axis view via a subcostal approach [15] using a curvilinear phased array probe (2–5 MHz) of Sono Site M-turbo (Fujifilm Sono Site, Inc., Bothell, USA). Then, variations in IVC diameter with respiration were assessed 2 to 3 cm distal to the right atrium using M-mode imaging generated at a medium sweep speed. The maximum expiratory diameter of the IVC (dIVC expiration) and its minimum inspiratory diameter (dIVC inspiration) were measured over the same respiratory cycle. IVCCI was calculated as a percentage using the formula IVCCI = (dIVC expiration – dIVC inspiration) × 100/dIVC expiration [16]. IVCCI was assessed by the same trained anesthesiologist who was blinded to postinduction hemodynamic measurements.

Anesthesia was induced using iv propofol 1.5–2 mg/kg and fentanyl 2 µg/kg. Endotracheal intubation was facilitated using cisatracurium 0.15 mg/kg. Anesthesia was maintained using isoflurane in an oxygen-air mixture based on MAC 1.25%. The patient was mechanically ventilated, and end-tidal carbon dioxide (ETCO_2_) monitoring started. The tidal volume was 6–8 ml/kg, and the respiratory rate was adjusted to achieve an ETCO_2_ of 30–35 mmHg. Ringer’s lactate was intravenously infused at a rate of 6 ml/kg/h.

Hypotension after induction of GA was considered if SAP dropped by more than 30% of the baseline value and/or mean arterial pressure (MAP) dropped to < 65 mmHg at any time after induction of GA until 15 min after endotracheal intubation and before the start of any surgical manipulations. If hypotension occurred, arterial blood pressure was measured every 3 min until hypotension subsided. A 250 ml crystalloid bolus was given and repeated as appropriate if MAP < 65 mmHg. Ephedrine 3 mg increments were given if hypotension persisted for ≥ 3 min.

At the end of the surgical procedure, anesthesia was discontinued, and muscle relaxation was reversed. The trachea was extubated when extubation criteria had been met.

### Data collection

Collected data included patient age, sex, BMI, ASA class, coexisting diseases, and the type of antihypertensive medication used. The IVCCI (primary outcome) was calculated based on the measurement before the induction of GA. Hemodynamics (HR, SAP, and MAP) were recorded as a baseline (before induction of anesthesia), after induction of GA and before endotracheal intubation and 5, 10, and 15 min after endotracheal intubation. The lowest SAP and MAP values after induction of general anesthesia were used to calculate the percentage of maximum change from respective baseline values.

### Sample size calculation

Sample size was calculated via Epi-info 7 assuming the mean of IVCCI was 50 ± 28.9% vs. 31 ± 12% in those who had hypotension vs. those who had not [11], power of the study 80% and CI 95%, the estimated sample was 153 patients.

### Statistical analysis

Continuous parametric data are presented as the mean ± SD and were compared using Student’s *t* test. Nonparametric data are presented as the median and interquartile range (IQR) and were compared using the Mann‒Whitney test. Qualitative data are presented as numbers and percentages and were compared using the chi-square test or Fisher’s exact test as appropriate. ROC curve analysis was used to assess the ability of IVCCI to predict hypotension after GA. The occurrence of hypotension after GA induction was considered the gold standard. The AUC was measured, and the best cutoff value was defined with associated sensitivity, specificity, positive predictivity (PPV) and negative predictivity (NPV).

A gray zone approach was used to define the range of uncertainty of IVCCI measurement values in predicting hypotension after GA. Gray zone values are values lying between values associated with 90% sensitivity and those with 90% specificity on their corresponding sigma curves of IVCCI. [17] IVCCI values were correlated with the percentage of maximum drop of SAP and MAP values after induction of GA from baseline (Pearson correlation).

## Results

**Fig. 1:**
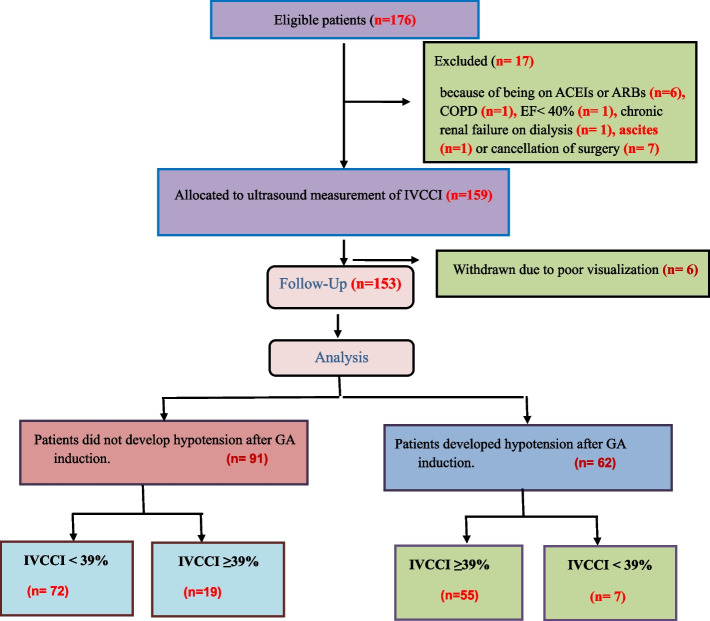
Study flowchart. ACEIs: angiotensin-converting enzyme inhibitors, ARBS: angiotensin receptor blockers, COPD: chronic obstructive pulmonary disease, EF: ejection fraction, GA: general anesthesia, IVCCI: inferior vena cava collapsibility index

 Of 176 eligible patients, 17 patients were excluded due to treatment with ACEIs or ARBs, depressed ventricular function, ascites, chronic renal failure on dialysis, COPD or cancellation of surgery. Six patients were withdrawn due to difficult visualization of the IVC. One hundred fifty-three patients completed the study, and their data were considered for statistical analysis (Fig. 1).

The mean patients' age was 46.88 y ± 7.58 standard deviation (SD). The mean BMI was 28.01 kg/m2 ± 2.66 SD. A total of 45.6% of patients were males, and 54.2% of patients were females. A total of 92.2% of patients were ASA II, and 7.8% of patients were ASA III. 34% of the studied patients had a history of DM, 5.2% had a history of ischemic heart disease (IHD), and 2.6% had a history of chronic atrial fibrillation (AF). A total of 67.3% of patients were on calcium channel blockers, 24.8% of patients were on β blockers, and 30.7% of patients were on diuretics. Most patients underwent elective craniotomy, lumbar fusion surgery, thyroidectomy, and radical hysterectomy (22.2%, 22.2%, 13.1% and 10.5%, respectively). Other surgical interventions included cervical fusion surgery, mastectomy, discectomy, nephrectomy, tympanoplasty, hemicolectomy, rhinoplasty, splenectomy, and Whipple operation.

The mean IVCCI for all patients was 40 ± 11%. The incidence of systemic hypotension after the induction of anesthesia was 40.5% among the studied population.

 There was no significant difference between patients who developed hypotension after the induction of anesthesia and those who did not regarding patient characteristics except for age. Patients who developed hypotension were significantly older than patients with no hypotension (*p* < 0.001). No significant difference was found between the two groups of patients regarding the type of antihypertensive medications used. The most frequent procedure associated with the development of hypotension after the induction of GA was radical hysterectomy (p 0.002). When planned for other surgical interventions, the number of patients who developed hypotension after induction of anesthesia was comparable to the number of patients who did not (Table 1).

**Table 1:** Characteristics of patients who developed hypotension after induction of anesthesia versus those of patients who did not

Variable		Hypotension (***n***=62)		No hypotension (***n***=91)		***P***
**Age (years)**	Mean ± SD.	50.11±8.46		44.67±6.04		<0.001*
**BMI (kg/m** ^**2**^ **)**	Mean ± SD.	28.13±2.89		27.93±2.49		0.65^**a**^
**Variable**		**No**	**%**	**No**	**%**	P
**Gender**	Male	31	50	39	42.8	0.38^**b**^
	Female	31	50	52	57.2	
**ASA**	II	59	95.2	82	90.1	0.25^**b**^
	III	3	4.8	9	9.9	
**Comorbidities**	DM	20	32.2	21	23.1	0.60^**b**^
	DM +IHD	2	3.2	5	5.5	
	DM+AF	1	1.6	3	3.3	
	IHD	0	0	1	1.1	
**Anti-hypertensive medications**	Calcium channel blockers	40	64.5%	63	69.2%	0.542^**b**^
	Β. Blockers	18	29%	20	22%	0.321^**b**^
	Diuretics	21	33.9%	26	28.6%	0.485^**b**^
**Surgical procedure**	Craniotomy	11	17.7	23	25.8	0.271
	Lumbar fixation	10	16.2	24	26.4	0.135
	Thyroidectomy	9	14.5	11	12.1	0.662
	Radical hystrectomy	**15**	**24.3**	1	1.1	**0.002**^**b**^
	Cervical fixation	6	9.7	6	6.6	0.548
	Mastectomy	3	4.8	8	8.8	0.527
	Discectomy	2	3.2	7	7.7	0.313
	Nephrectomy	3	4.8	3	3.3	0.687
	Tympanoplasty	1	1.6	5	5.5	0.402
	Hemicolectomy	2	3.2	0	0	0.163
	Rhinoplasty	0	0	1	1.1	1.000
	Splenectomy	0	0	1	1.1	1.000
	Whipple operation	0	0	1	1.1	1.000

*SD* Standard deviation, *DM* Diabetes mellitus, *IHD* Ischemic heart disease, *AF* Atrial fibrillation

^a^Independent t test ^b^Chi square test

**Fig. 2:**
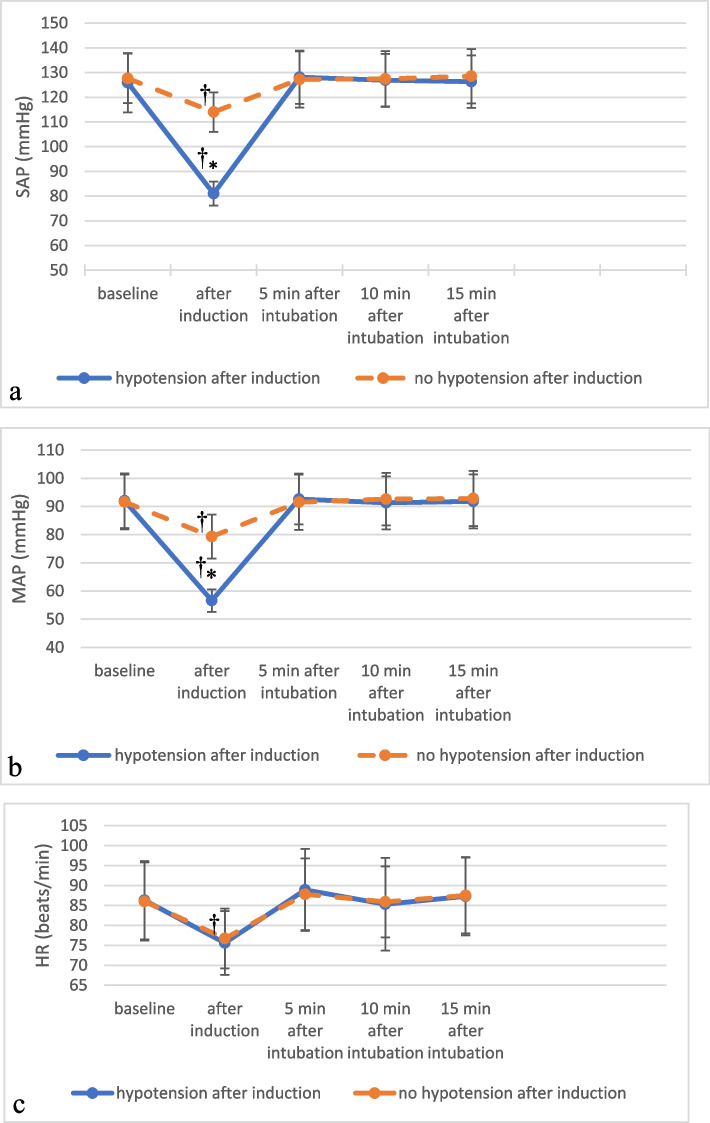
(**a**) systolic arterial pressure (SAP), (**b**) mean arterial pressure (MAP) and (**c**) heart rate (HR) of patients who developed hypotension versus those of patients who did not at different measuring points. Values are presented as the mean ± SD. * significant difference between both groups. ^†^ Significant difference from baseline value

 The significantly lowest SAP, MAP and HR values of all patients were those recorded after induction of GA and before tracheal intubation compared to baseline values. After intubation, the average SAP, MAP and HR values were comparable to those recorded as baseline values (*p* ≥ 0.05) (Fig. 2).

Baseline SAP and MAP values and those recorded after intubation were comparable between patients who developed hypotension after induction of GA and those who did not (Fig. 2a, b). Of the 62 patients who developed hypotension, 4 patients (6.5%) needed crystalloid bolus, and 10 patients (16%) received crystalloid bolus and ephedrine increments. There was no significant difference between patients who developed hypotension after induction of anesthesia and those who did not regarding HR at all measuring points (*p* ≥ 0.05) (Fig. 2c).

**Fig. 3:**
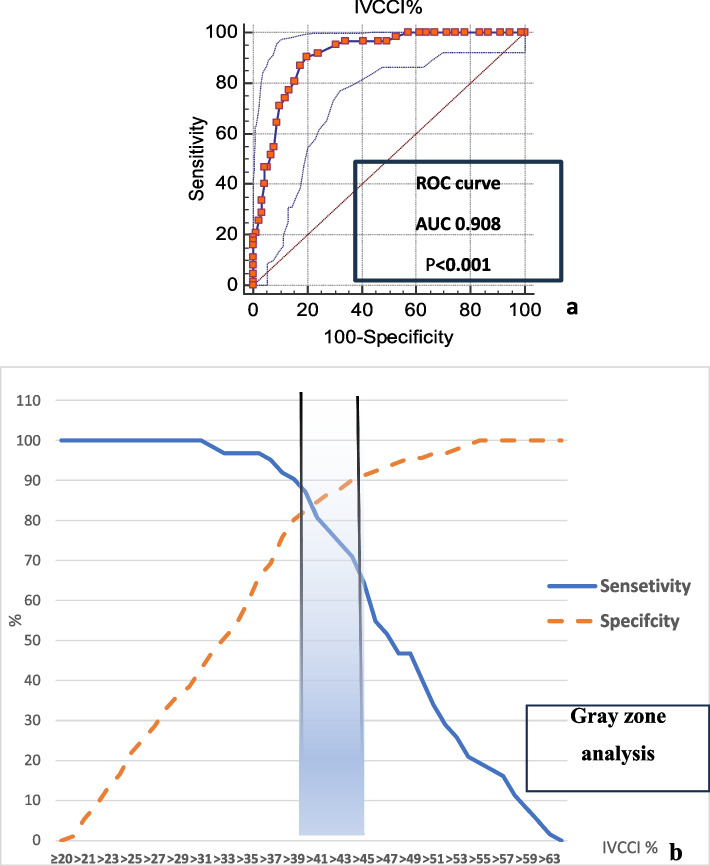
The performance of IVCCI % as a predictor of hypotension after induction of GA in hypertensive patients (**a**): ROC curve, (**b**) gray zone analysis. IVCCI: Inferior vena cava collapsibility index; GA: General anesthesia AUC: Area under curve; ROC: Receiver operating characteristic

 The average IVCCI of patients who developed hypotension was significantly higher than that of patients who did not (49 ± 8 vs. 33 ± 8% respectively) (*p* < 0.001). An IVCCI > 39% can significantly predict hypotension after the induction of GA (*P* < 0.001). Gray zone analysis revealed that the area of uncertainty of the diagnostic test lies at IVCCI values from 39 to 45%. This gray zone included 21 patients (13.7% of all patients). Seven patients (4.6% of all patients) were hypotensive, while the IVCCI value was < 39% (Table 2; Fig. 3).

**Table 2:** The performance of IVCCI % as a predictor of hypotension after induction of GA in hypertensive patients

**Cut off**	**AUC (95% CI)**	**Sens. (%)**	**Spec. (%)**	**PPV (%)**	**NPV (%)**	**+LR**	**-LR**	**Accuracy (%)**	**P**
**>0.39**	**0.908 (0.851-0.949)**	**90.32**	**80.22**	**75.66**	**92.4**	**4.57**	**0.12**	**84**	**<0.001**

*IVCCI* Inferior vena cava collapsibility index, *GA* General anesthesia, *AUC* Area under curve, *CI* Confidence interval, *Sens* Sensitivity, *Spec* Specificity, *PPV* Positive predictive value, *NPV* Negative predictive value, *+LR* Positive likelihood ratio, -*LR* Negative likelihood ratio

There was a significant positive correlation between IVCCI% before induction of GA and the percentage of maximum SAP drop after induction of GA (*r* = 0.685) (*P* < 0.001) as well as between IVCCI% and the percentage of maximum MAP drop after induction of GA (*r* = 0.678) (*P* < 0.001).

## Discussion

The current study revealed that IVCCI > 39% before induction of GA can predict the onset of hypotension after induction of GA for noncardiac surgery in hypertensive patients not treated with ACEIs or ARBs. The gray zone existed for IVCCI between 39 and 45% and included 13.7% of the studied patients. IVCCI before induction was found to strongly correlate with the maximum percentage of SAP or MAP drop after induction of GA. The incidence of postinduction hypotension was 40.5% among the studied hypertensive patients.

It was shown that even short periods of intraoperative hypotension (1–5 min) can be associated with increased perioperative morbidity in cardiac and noncardiac surgeries [5, 18]. Therefore, the prediction of postinduction hypotension is essential to select patients at risk who may need further preoperative volume administration. The incidence of hypotension for 15 min after intubation was chosen as an outcome measure to truly represent anesthesia-related hypotension. Hypotension under GA has several reasons and contributors, amongst them, dehydration is the most frequent. So, hypotension may simply be avoided by optimization of preoperative fluid status in predicted patients [19].

IVCCI needs little echocardiographic experience to assess, in addition to being a feasible, noninvasive, and cost-effective tool for assessing intravascular volume and fluid responsiveness in critically ill patients [10, 20].

Similar to current findings, IVCCI > 38–50% can predict hypotension after induction of GA by different accuracies (AUC 0.648–0.959) in mixed patients’ population with or without cardiovascular diseases (CVD) undergoing either cardiac and non-cardiac procedures [6, 11, 21].

The gray zone approach by Zhang and Critchley revealed an area of uncertainty of IVCCI values between 38% and 43%, including 12% of all studied patients. For patients with CVD, a wider gray zone for IVCCI was found in patients with CVD (29–43%), indicating less reliability of IVCCI in the prediction of postinduction hypotension in patients with CVD. Our findings revealed a narrower gray zone of IVCCI for the prediction of postinduction hypotension (39–44%) and hence higher reliability in hypertensive patients. Similar to the current findings, Zhang and Critchley found a significant correlation between IVCCI and the percentage of maximum drop in MAP after induction of GA (*r* = 0.46 vs. *r* = 0.678 in the current study) [11].

In contrast to the current finding, Zhang and associates demonstrated poor performance of IVCCI (best cutoff value 50%) before induction in predicting hypotension after induction of GA in hypertensive patients (AUC 0.523 vs. 0.908 in the current study), while they found a comparable predictivity in normotensive patients (AUC 0.896). The different findings from the current study may be due to different sample sizes (51 vs. 153 hypertensive patients in the current study), different outcome definitions (based on MAP only in Zhang’s study vs. considering both SAP and MAP in the current study) or the state of hypertension control in the included patients (heterogenous population in Zhang study vs. controlled hypertensive patients in the current study) [22].

Agarwal and associates demonstrated poor accuracy (53%) of IVCCI (best cutoff value ≥ 63.3%) in the prediction of postinduction hypotension in the general population (AUC 0.568, sensitivity 31% and specificity 84%). The difference between Agarwal’s study and the current study may be due to different studied populations (general population versus controlled hypertensive population in the current study) or older age of patients in the current study [23].

Mohammed and associates also showed poor accuracy of IVCCI ≥ 46% in predicting hypotension after induction of GA in a young healthy adult population (AUC 0.51) and found no correlation between IVCCI and maximum drop in MAP. The differences from current findings may be due to different studied populations, different incidences of postinduction hypotension (19.3% in the Mohammed study vs. 40.5% in the current study) or different sample sizes [24].

Patients who developed hypotension in the current study had higher IVCCI, and they were older in age. Increasing age can be associated with increased respiratory variability of IVC [25]. Being at higher risk of dehydration, the older population may have higher IVCCI as a pathophysiologic response rather than just a confounder [6]. The hypovolemia of those older patients may be aggravated by the higher proportion of planned radical hysterectomy in this group of patients. radical hysterectomy patients were suffering from preoperative vaginal bleeding and some of them required preoperative mechanical bowel preparation. Considering the interaction of physiological factors affecting respiratory variation in IVC diameter, such as venous compliance, intraabdominal pressure and volume status, the interpretation of IVCCI values should be interpreted within the patient clinical context [25].

This current study was limited first, as it did not include measurement of maximum IVC diameter as another potential predictor of hypotension after induction of GA. Second, the variability of operative interventions studied. Third, patients on ACEIs or ARBs medications were excluded from the study to avoid their high vasodilatory effect as a confounder and so the results of current study cannot be applied to such patients’ population.

Ultrasonographic assessment of IVCCI still can be used preoperatively as a point of care tool to predict the onset of hypotension after induction of GA especially in patients at high risk of developing hemodynamic instability. Szabó and associates succeeded in decreasing the incidence of early intraoperative hypotension by using both preoperative IVCCI and pulmonary ultrasound to guide preprocedural fluid therapy [26]. Ultrasonographic IVC assessment, to guide fluid administration before spinal anaesthesia, can reduce the incidence of spinal-anesthesia-induced hypotension [27, 28]. Further studies are recommended to investigate whether preoperative IVCCI-guided fluid management can decrease the incidence of postinduction hypotension.

## Conclusion

An inferior vena cava collapsibility index > 39%, before anesthetic induction, can be a simple noninvasive reliable predictor of hypotension after induction of GA for hypertensive patients not treated with ACEIs or ARBs and undergoing noncardiac surgery. For the same population, there was a strong positive correlation between IVCCI before the induction of GA and the maximum drop in either SAP or MAP after the induction of GA.

## Data Availability

The data used and analyzed during our study are available from the corresponding author upon reasonable request.

## Abbreviations

ACEIs: Angiotensin-converting enzyme inhibitors
ARBs: Angiotensin receptor blockers
AUC: Area under curve
COPD: Chronic obstructive pulmonary disease
CVD: Cardiovascular diseases
dIVC: Diameter of the inferior vena cava
DM: Diabetes mellitus
ETCO2: End-tidal carbon dioxide
GA: General anesthesia
HR: Heart rate
IHD: Ischemic heart disease
IVCCI: Inferior vena cava collapsibility index
MAP: Mean arterial pressure
NPV: Negative predictive value
PPV: Positive predictive value
RASS: Richmond Agitation Sedation Scale
ROC: Receiver operating characteristic
SAP: Systolic arterial pressure
